# A New Methodology for Studying Dynamics of Aerosol Particles in Sneeze and Cough Using a Digital High-Vision, High-Speed Video System and Vector Analyses

**DOI:** 10.1371/journal.pone.0080244

**Published:** 2013-11-27

**Authors:** Hidekazu Nishimura, Soichiro Sakata, Akikazu Kaga

**Affiliations:** 1 Virus Research Center, Clinical Research Division, Sendai Medical Center, Sendai, Japan; 2 Takasago Thermal Engineering. Co., Ltd., Tokyo, Japan; 3 Graduate School of Engineering, Osaka University, Osaka, Japan; Lovelace Respiratory Research Institute, United States of America

## Abstract

Microbial pathogens of respiratory infectious diseases are often transmitted through particles in sneeze and cough. Therefore, understanding the particle movement is important for infection control. Images of a sneeze induced by nasal cavity stimulation by healthy adult volunteers, were taken by a digital high-vision, high-speed video system equipped with a computer system and treated as a research model. The obtained images were enhanced electronically, converted to digital images every 1/300 s, and subjected to vector analysis of the bioparticles contained in the whole sneeze cloud using automatic image processing software. The initial velocity of the particles or their clusters in the sneeze was greater than 6 m/s, but decreased as the particles moved forward; the momentums of the particles seemed to be lost by 0.15–0.20 s and started a diffusion movement. An approximate equation of a function of elapsed time for their velocity was obtained from the vector analysis to represent the dynamics of the front-line particles. This methodology was also applied for a cough. Microclouds contained in a smoke exhaled with a voluntary cough by a volunteer after smoking one breath of cigarette, were traced as the visible, aerodynamic surrogates for invisible bioparticles of cough. The smoke cough microclouds had an initial velocity greater than 5 m/s. The fastest microclouds were located at the forefront of cloud mass that moving forward; however, their velocity clearly decreased after 0.05 s and they began to diffuse in the environmental airflow. The maximum direct reaches of the particles and microclouds driven by sneezing and coughing unaffected by environmental airflows were estimated by calculations using the obtained equations to be about 84 cm and 30 cm from the mouth, respectively, both achieved in about 0.2 s, suggesting that data relating to the dynamics of sneeze and cough became available by calculation.

## Introduction

Microbial pathogens in the secretion of the respiratory tract of respiratory infectious disease patients are brought into the environment through sneezing or coughing, thus rendering the diseases contagious. The strong airflow of sneeze and cough blows on the respiratory tract surface, tearing off the secretions and producing biomist particles of various sizes. Importantly, the particles sometimes contain pathogens and often directly contact other individuals by motion of the mist itself, driven by the airflow of cough and sneeze, or indirectly reach the respiratory tract(s) of other individual through inhaling while floating in the environmental air after losing their initial speed due to air friction. Therefore, for controlling the spread of infectious respiratory diseases, it is very important to understand the movement of the bioparticles caused by coughing and sneezing.

In the early 1940s, Jennison et al. took serial photo images of a sneeze of an adult male subject using a high-speed camera in an attempt to visualize the bioparticles expelled during sneezing. However, because of the technical limitations of that time, they were only able to show the movement of the particles with diameters large enough to fall to the ground at speeds of milliseconds. Nonetheless, they were able to estimate that the maximum distance spanned by the droplets was 2–3 feet at most, and that their initial speed was at least 152 ft/s [1].

Zhu et al. attempted to visualize the movement of cough droplets, assuming that they were saliva bioparticles, by using a pulse laser beam coupled with a digital video camera. However, in their study, wheat flour was used as the tracer for the visualization and was expelled with the cough from the subject’s mouth. As a result, only very large, artificial, solid particles flying long distances and with strong inertia were analyzed [2].

Recently, Tang et al. reported real-time visualization of cough airflow by schlieren analysis [3]. Kwon et al. employed particle image velocimetry (PIV) to trace the movement of airborne particles. Using an acrylic indoor chamber containing atomized oil, a thin sheet of laser beam, and a charge-coupled device camera [4], they were able to detect the motion of cough airflow, recognized as the difference of density from the surrounding air or as movement of the fine oil particles. There are no studies that directly analyze the motion of the particles themselves, with the exception of that by Chao et al. Using PIV with a laser sheet positioned before the mouth and without the aid of oil mist, Chao et al. described the movement of cough particles close to the mouth [5].

Here, we report for the first time the analysis of the dynamic of sneeze bioparticles not only in the mouth vicinity but for the whole sneeze cloud containing fine particles invisible to the naked eye, using a healthy volunteer as a model. The analysis was made possible by an image-capturing system using a digital high-vision, high-speed video camera equipped with a computer system. The obtained video image of the particles was subjected to the vector analysis to obtain an approximate equation of a function of elapsed time for their velocity. Thus, we could introduce a novel methodology to calculate the direct reach of the sneeze bioparticles. This methodology was also applied for a cough, using a smoke exhaled with the cough after smoking one breath of cigarette by a healthy adult volunteer, by which the dynamics of the fine cough bioparticles invisible with our system could be estimated by using microclouds visible in the smoke as aerodynamically acceptable surrogates for the particles.

## Materials and Methods

This study was approved by the ethical committee of Sendai Medical Center, and written informed-consent was obtained from volunteers involved in this study. The subjects of the photographs had given written informed consent to publication of their photograph, as outlined in the PLOS consent form.

### Imaging of sneeze

A male volunteer in his early 20s was invited under informed consent to a broadcasting studio to sneeze artificially by stimulating the mucus membrane of his nasal cavity. The stimulation was conducted by the volunteer himself using a roll obtained by twisting a sheet of tissue paper. The temperature and relative humidity (RH) of the studio were 27°C and 15%, respectively. The image of the sneeze was taken and recorded by a digital high-vision, high-speed video system (Japan Broadcasting Corporation, Tokyo, Japan). The images were taken from the lateral position, vertical to the direction of the sneeze’s main forward movement, with lighting from an appropriate position in a dark field. The enhanced images were obtained by binary image processing of the original image using the function available with the video system.

### Imaging of cough

The biomist cloud of the cough was substituted with cigarette smoke. A smoker male volunteer in his 30s, who occasionally shows cough symptoms, was asked to smoke one breath of cigarette and to voluntarily cough in a laboratory under informed consent. The temperature and RH of the laboratory were 27°C and 50%, respectively. The image of the movement of the smoke exhaled with the cough was taken and recorded by an digital high-speed video system (nac Image Technology Inc.,Tokyo, Japan) from the lateral position, in the same way as the imaging of the sneeze was conducted.

### Vector analysis

Vector analysis of the sneeze was performed on the movement of each biomist particle or cluster of multiple particles [i.e., the particle cluster (PC)], recognized as a particular granular signal in the video image. Vector analysis of the cough was performed on small smoke clouds, microclouds (MCs), probably composed of very fine smoke particles. The MCs could be separately identified by their different tone densities of white color and were used as substitutes for the cough bioparticles.

The analog images of the sneeze and cough from 0 to 0.4 s after release were converted every 1/300 s into 120 frames of digital images with a 640×400 pixel resolution. Each image brightness signal was tracked automatically during the 1/300 s using image processing software that employed PIV, to which the successive abandonment method was applied for improvement [6], [7]. The information obtained from the movement of particles or the MCs were displayed as vectors with color graduation on the basis of their velocity levels. The dimensions of the reference area were set at 21×21 pixels, making the spatial resolution for the measurement about 4.4 cm. Assuming a 1/10 pixel error caused by the interpolative calculation of the moving distance on a subpixel scale [8], the maximum error in the measurement of the velocity vector was calculated to be 6.3 cm/s.

## Results

### Image analysis of the movement of particles in the sneeze

**Figure 1:**
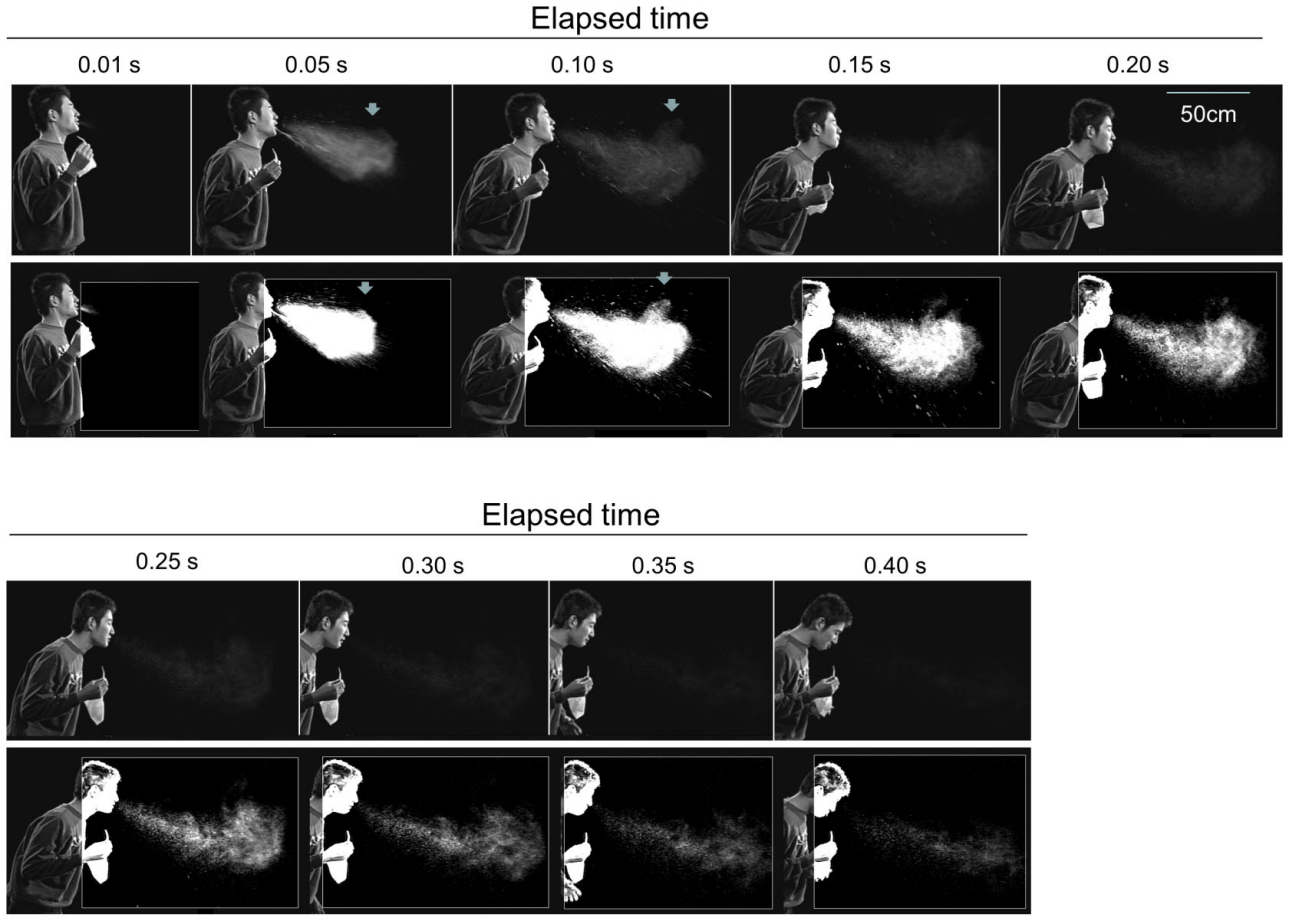
Serial photographs of a sneeze extracted from the video image. Original (upper rows) and enhanced (lower rows) images of the sneeze of a healthy adult male volunteer. The photographs were extracted from the video image recorded by a digital high-vision, high-speed video system at 0.01 s and every 0.05 sec. The mist of the sneeze advanced forward as a mass, associated with gradual diffusion and fading followed by disappearance. A part of the mist cloud looked swirled in the peripheral area (arrows).

An image of a sneeze of a 180 cm tall healthy male volunteer in his early 20s was taken and recorded using an HDTV high-speed video system in a closed studio (temperature 27°C, RH 15%) under calm conditions with a maximum air velocity of less than 20 cm/s. The images were gleaned every 0.05 s and arranged chronologically. In Fig. 1, the upper rows represent the unprocessed original images, while the lower rows correspond to their respective images enhanced by binary image processing. The sneeze mist mass advanced gradually diffusing as a whole for 0.3 s, then it seemed to fade until it disappeared after 0.40 s. However, the enhanced images suggest that each particle or PC, recognized as a particular granular signal, decreased in size, but they were still present even after 0.40 s. It was also clear that the largest droplets fell with a very high speed and disappeared from the image within 0.20 s. It is interesting to note that a part of the mist cloud began to swirl in air after 0.05 s, at the level of the upper peripheral area about 50 cm horizontally from the mouth. This behavior was probably due to the turbulence of the expelled air and caused the particles to disperse in that area (Fig. 1).

**Figure 2:**
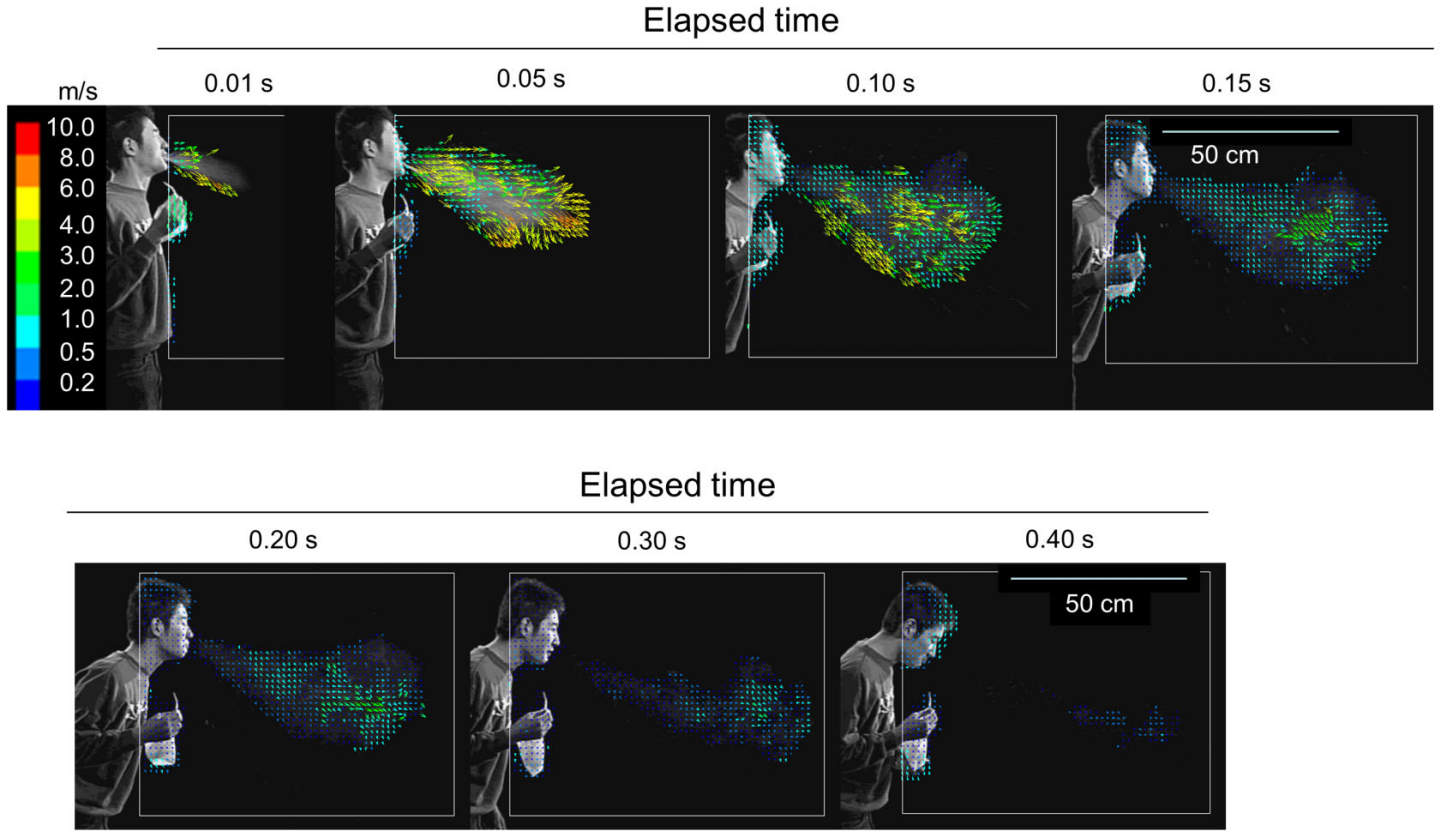
Vector analysis of particles movement in the sneeze. The video image was converted to digital images collected every 1/300 s. Each brightness pattern of the aerosol particles or particle clusters, recognized as a particular granular signal, was automatically traced during 1/300 s using image processing software. The vectors are color graduated according to their velocity levels.

**Figure 3:**
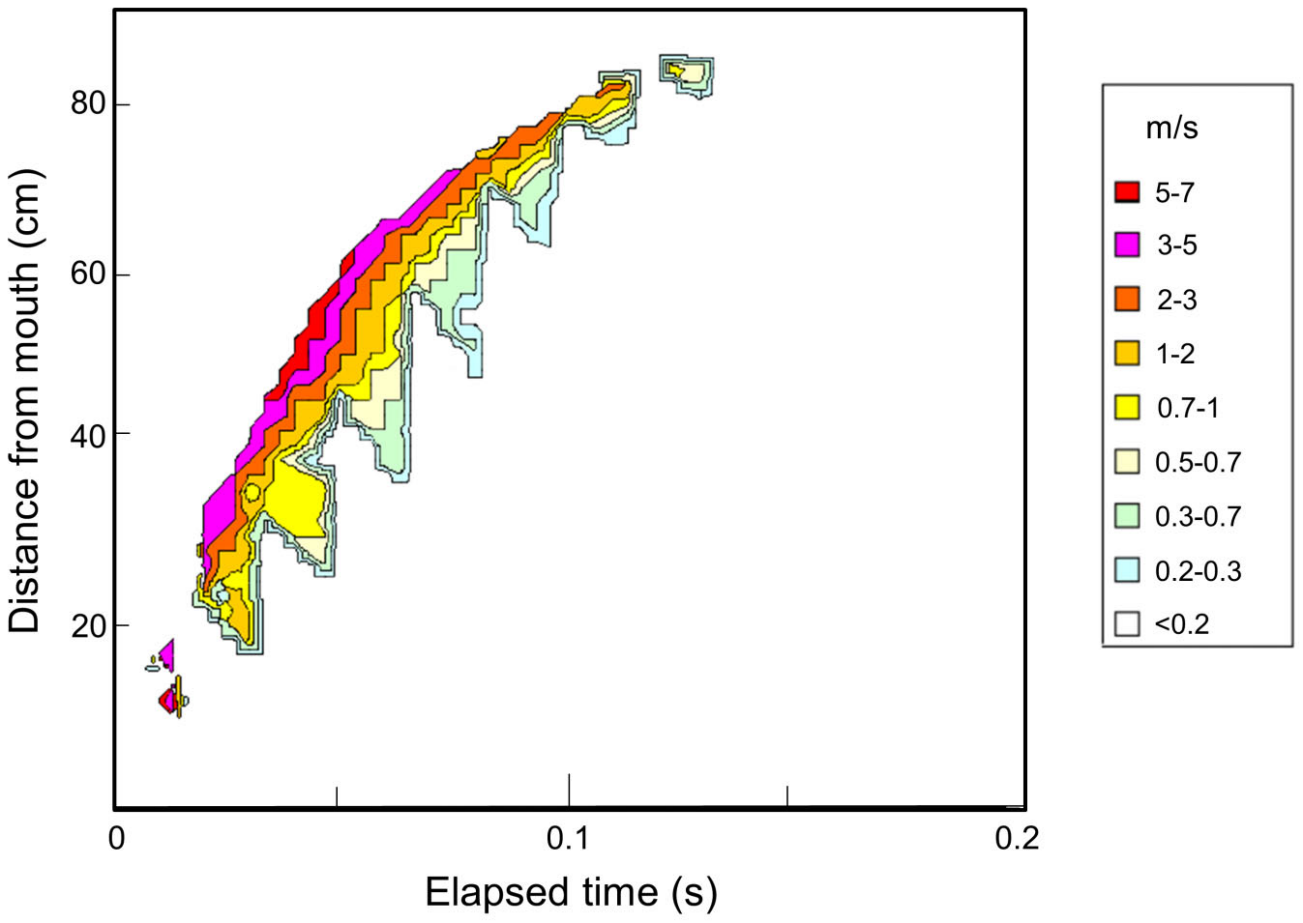
Velocity distribution of particles moving forward as a mass in the sneeze. Information on the position and horizontal velocity of each particle/particle cluster was extracted from the results of the vector analysis of the sneeze and is plotted along with the elapsing time on a two-dimensional graph. The velocity levels are expressed by color graduation.

Vector analysis on the image of the sneeze biomist, excluding the fast-falling large droplets, of which size were estimated to be more than 300 µm in aerodynamic diameter by calculation based on the settling velocity of the particles (data not shown), was performed. The mist particles in the sneeze had an initial velocity of greater than 6 m/s, which decreased as they moved forward. The particles almost lost their momentum at 0.12–0.15 s, and thereafter began to diffuse (Fig. 2). The distribution of the absolute velocities of the vectors was displayed in Fig. 3 to capture the individual movements easily as a mass. By extracting information on the position and velocity of each vector, the vectors were plotted on a two-dimensional graph bearing distance and time on the axes. The velocity was expressed by color graduation. It was immediately clear that during the first 0.08 s, the velocity of the particles located at the distal end (i.e., 75–80 cm from the mouth) was highest in the mist cloud, and thereafter, decelerated to become slower than the particles found at the inner part of the mist mass, and finally almost lost velocity by 0.15 s, about 85 cm from the mouth (Fig. 3).

### Image analysis of movement of particles in the cough

For visualizing the movement of the cough particles, we first simply tried to record a high-speed video image in the same way as that for the sneeze, but this attempt was not successful. We repeatedly tried changing the experimental conditions, such as the direction of the light beam and different kinds and strengths of the light source including laser scattering. However, we could only take a faint image of the mist, which was not sufficient for further analyses. We thought that the problem was not the dimension of the particles compared with those in the sneeze, but the fact that the amount of particles in the cough is much less than that in the sneeze [9], [10] and the particle concentration is too low to visualize them. Consequently, considering that the particle concentration in cigarette smoke cough is much higher than in natural cough and that smoke is very well visible, we eventually took the video image of the smoke cough.

In aerodynamics, it is well known that the movement of fine particles, the size range from submicron to 10 µm level, follows that of the air that carries them, and the smaller the particles, the shorter the time lag necessary for the particles to follow it [11]. The size of cigarette smoke particles exhaled from the smoker was reported to be submicron size [12] and small enough to assume that their movement represents the movement of the airflow.

On the other hand, the dimensions of the biomist particles derived from coughing were reported to be mainly in the range from submicron to several micrometers as well [10],[13]–[15] in the dried condition, while larger sizes (0.6–16 µm: median 8 µm) were also reported [5],[15] in the condition without evaporation of the water from the particle. We thought that most of the cough particles are in the dried condition and at least in the size range that move with the airflow of the cough, when a certain time has elapsed from the release in the environmental air. Therefore, the above-mentioned relationship of the fine particles with the airflow could be applied to those in the cough as well, meaning that the movement of the smoke particles and airborne cough particles would be almost the same in the airflow of the cough.

**Figure 4:**
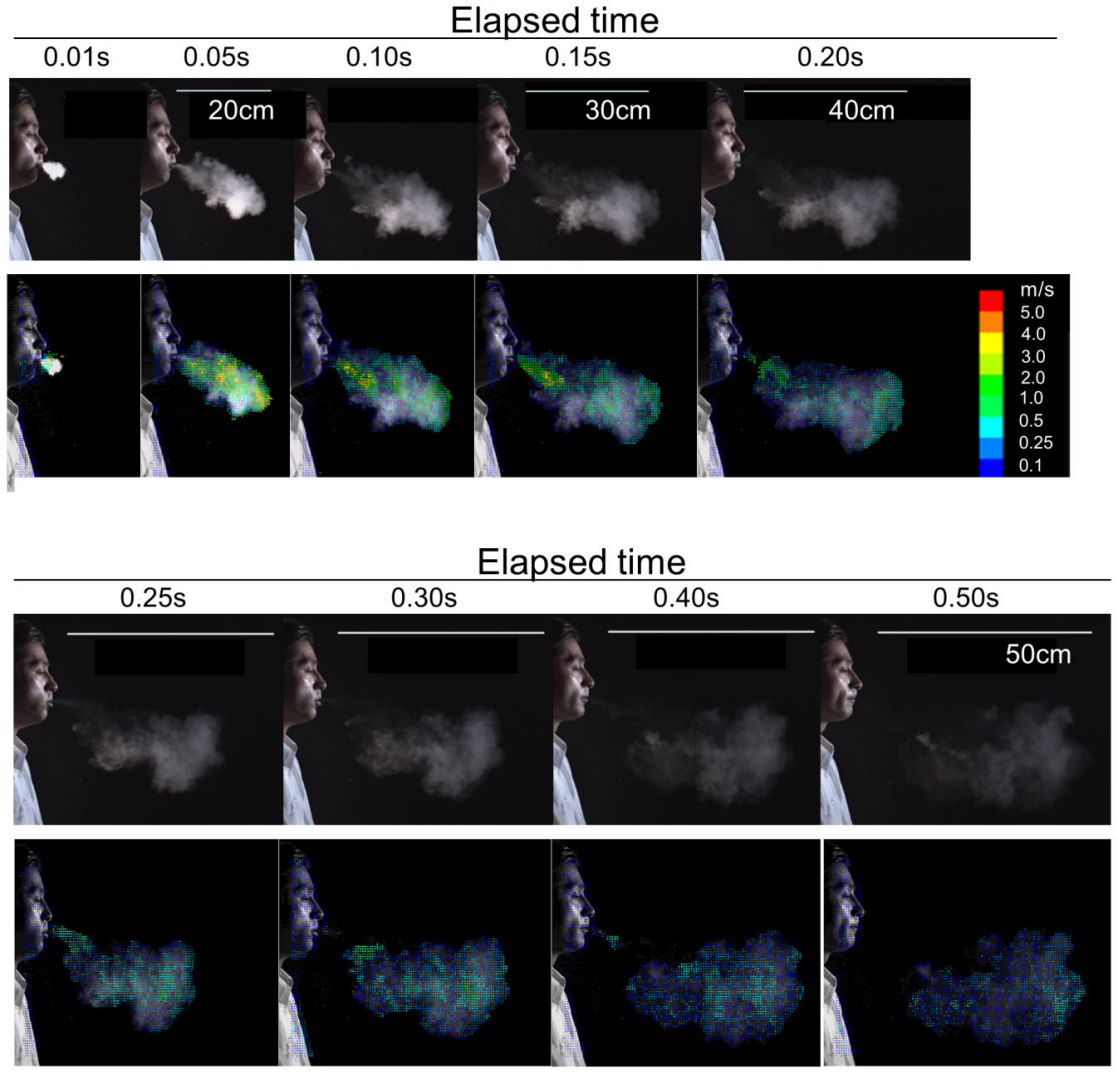
Image of cough movement represented by smoke cough. Image analysis of the cough of a smoker healthy adult male volunteer after one breath of smoke. The image of the natural cough was substituted with that of cigarette smoke used as the tracing marker. The photographs were extracted from the video image using a digital high-vision, high-speed video system (upper rows). Vector analysis was performed on the microcloud identified with various densities of white tones of the smoke (lower rows), as it was done for the particles/particle clusters of sneeze.

The upper panel of Fig. 4 shows the unprocessed original images of a cough smoke mass of a 170 cm tall healthy male volunteer in his early 30 s coughing after one breath of cigarette smoke in a laboratory. The photographs are arranged chronologically at intervals of 0.05 s.

Although the size of individual cigarette smoke particle is submicron level by optical measurement and hardly recognizable even by our video system, it is possible to actually observe the movement of individual MCs in the smoke mass, recognized as small white tones of the smoke with various densities. It is known that the inhaled cigarette smoke particles cause the interparticle hydrodynamic interactions, coagulation or hygroscopic growth inside the respiratory tract and the coagulated particles behave aerodynamically as if they are particles of 6.5–7.1 µm-size [16], [17]. We considered that the MCs might be such particles or their cluster large enough for visual recognition by the video system and could behave aerodynamically same with fine bioparticles of less than that size level and, therefore, could be good visible surrogates for the bioparticles in the cough in the analysis of the dynamics of the particles [13]–[15]. Therefore, their movement was subjected to the vector analysis (Fig. 4).

The horizontal components of the velocities of MCs were initially higher than 5 m/s. For the first 0.05 s, the highest velocities were located at the forefront of the smoke cloud mass, which was moving forward as a whole. Thereafter, the velocity of the forefront clearly decreased probably owing to air friction and gradually became inhomogeneous after 0.10 s at about 25 cm from the mouth. Each MC began to diffuse.

### Estimation of momentum-driven maximum direct reaches of sneeze, cough particles, and cough airflow

Although it would be interesting to determine the distance that particles released by sneezing and coughing are able to reach, this kind of measurement is very difficult because the natural convection current always exists in every indoor space, as the insensible airflow even when it is apparently calm and it affects the movement of the mist particles

**Figure 5:**
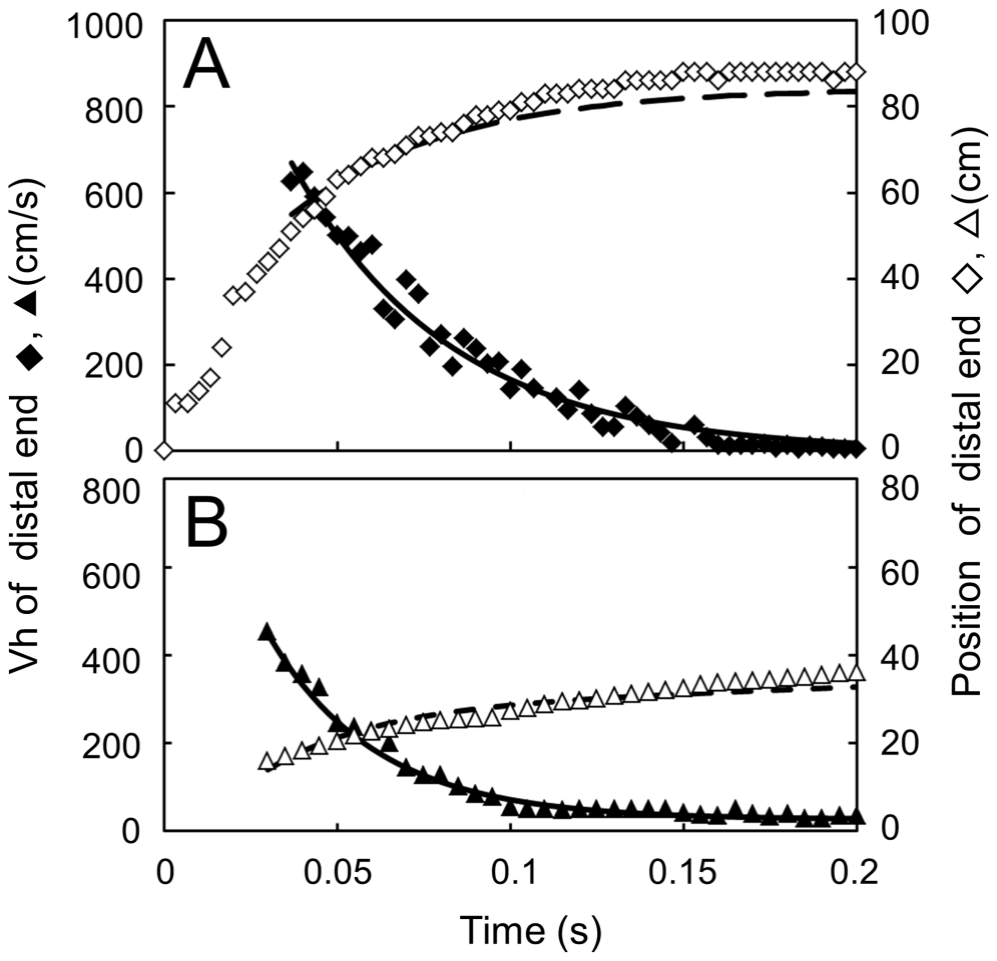
Position and velocity of particles at the distal margin of the sneeze and cough. Measured maximum horizontal velocity Vh (♦,▴) and its position: horizontal distance h, from the mouth (**◊**,△) of the sneeze (A) and cough (B). Values for Vh (solid line) calculated from the approximate equations for the sneeze (I) and for the cough (II), and their relative positions acquired by integration of Vh (dotted line) with respect to the elapsed time t after release.

To determine this distance by calculation without effect of the convection current, a graph showing the chronological correlation between the horizontal velocities of the particles located at the distal end of the mist mass in the sneeze and the elapsed time (time-Vh graph) was plotted (Fig. 5A). The vectors relative to individual particles or PCs located at the margin of each 21×21 pixel reference area of the mist mass was chosen, and the data of their horizontal component and distance from the mouth were plotted along the time axis. Before 0.04 s, the images of the particles were too dense; therefore, identification of the motion of individual particles was not accurate, and only the data from 0.04 to 0.20 s were used in this analysis.

Based on this analysis, we extrapolated the approximate equation describing the advancement of the front line of the sneeze (I), following which the horizontal velocity of the particles depends on the natural base exponential function of the elapsed time.

Vh  =  1500 exp(-22t) (I)

where Vh represents the horizontal velocity (cm/s) of particles or PCs at the distal end of the sneeze mist mass and t (s) is the time after the release from the mouth.

The velocity of natural convection current that exists as the insensible airflow is generally believed to be about 15 cm/s on average [18]. We postulated that the time when the particles lost their momentum corresponded to when their velocity reached the level of this current. Entering the value Vh  =  15 cm/s into the approximated equation leads to t  =  0.209 s. This means that the particles at the distal end of the mist cloud had lost their momentum about 0.21 s after the release in the sneeze. According to the result of the integral calculation from equation (I), their position corresponded to 84 cm from the mouth. Thereafter, the particles and PCs appeared to be dispersed, probably floating in the convection airflow of the room (Fig. 2).

For smoking cough, as it is evident from the downward convex appearance of the curve until 0.05 s from the release, the time-Vh graph in Fig. 5B shows that Vh had an initial value of greater than 5 m/s and received a strong negative acceleration until about 0.05 s. The movement of MCs in the front margin of the smoke mass drastically decreased in that time range up to 20 cm from the mouth, while after 0.10 s, the velocity gradually decreased and reached an apparently plateau value of about 30 cm/s at about 0.15 s. This value is higher than that of the sneeze recorded above. A possible reason for this result might be a stronger environmental airflow in the laboratory where the video was taken: one-directional air flow of 0.2–0.3 m/s along the cough flow due to air-conditioning convection.

Based on the data from the smoke cough time-Vh graph from 0.03 to 0.20 s, we extrapolated another approximate formula (II) similar to that of the sneeze.

Vh  =  1100 exp(–32t)+27 (II)

Entering the plateau value, Vh  =  30 cm/s, we obtained t  =  0.18 s. After solving the integral calculation of formula (II) from 0.03 to 0.18 s, it was suggested that the horizontal distance where cough particles had lost the momentum given by the cough was about 30 cm from the mouth.

Moreover, while the direct reach of the particles was estimated as described above, using the aerodynamic relationship between the movement of the airflow and fine particles [8], it would be equally possible to know the movement of the airflow through that of the fine particles being carried in it. Thus, the direct reach of the cough airflow, without the effect of the convection flow, was estimated using the above equation (II) by replacing the particles with the airflow. As a result, the maximum direct reach of the airflow of the cough of this subject was about 30 cm from the mouth, achieved at 0.18 s after release.

## Discussion

The study presented here is important in that the movement of particles in the human sneeze was visualized and analyzed, including both the large droplets that fall down in a very short time and tiny biomist particles that float in the air.

Concerning our analysis on the cough, owing to our technological limitations, initially we could not directly see the individual bioparticles that were assumed to be contained in it. The difficulties were overcome using particles of smoke MCs contained in the cough after cigarette smoking. Since particles of the exhaled smoke are small enough to float in the air without being affected by gravity [12], the movement of the MCs is considered to be the same in the cough as that of majority of natural cough mist particles with submicron or a few micron sizes [10],[13]–[15]: owing to the general assumption in aerodynamics that the movement of fine particles follows that of the airflow that carries them [11], the movement of the natural cough particles and smoke MCs could be considered almost the same in the cough airflow. This assumption enabled estimation of the movement of the invisible cough bioparticles. Based on the same reversed general assumption, we could also estimate the movement of the airflow of the sneeze and cough.

Tang et al. visualized the airflow of the cough of healthy white volunteers using schlieren imaging, with which the movement of the air mass could be detected as that of different thermal density from the surrounding air. Exploiting “schlieren PIV” analysis, they reported that the maximum velocity of the cough particles was greater than 8 m/s [3]. Kwon et al. used PIV with atomized oil in the air and reported that the initial velocity of cough flow was 15.3 m/s and 10.6 m/s for males and females, respectively [4]. In addition, Chao et al. used PIV without oil mist and reported initial velocities of 13.2 m/s and 10.2 m/s for males and females, respectively [5].

Although our study revealed that the initial velocity was greater than 6 m/s, specific velocities could not be reported owing to the limitations of our methodology: measuring the velocity of individual particle/PCs/MCs immediately after their release was impossible because their concentration was too high. However, our results did not contradict previous findings, meaning that almost the same conclusions were achieved using a different approach.

In our study, three major differences between the human sneeze and cough have been demonstrated: 1) the reach of the sneeze was three times longer than that of the cough; 2) the velocity of the sneeze after 0.05 s was about three times higher than the cough, and 3) in contrast to the sneeze, the velocity of the cough drastically decreased at about 0.05 s after the release. All these differences can probably be attributed to a difference in the blowing energy between sneeze and cough.

However, because our study was performed with only one subject for both sneezing and coughing, we cannot confirm whether our results were accidental. The approximate formulas extrapolated are specific for the subject under study and might be different for other individuals. In this respect, we are only proposing models and analysis options. It is important to test these procedures on many subjects, including individuals of different genders, races, age groups, body types, and health status as well as in various environmental conditions. In addition, in the context of the transmission of respiratory infections such as influenza, studies on individuals who contracted the disease as well as on healthy subjects would be important for infection control.
